# Influence of vertical growth pattern on masseter muscle morphology: evidence from cephalometric and ultrasound assessment in eighteen growing subjects

**DOI:** 10.3389/fdmed.2025.1748744

**Published:** 2026-01-07

**Authors:** Gianna Dipalma, Alessio Danilo Inchingolo, Mariafrancesca Guglielmo, Daniela Di Venere, Francesco Inchingolo, Andrea Palermo, Grazia Marinelli, Angelo Michele Inchingolo

**Affiliations:** 1 Department of Interdisciplinary Medicine, University of Bari “Aldo Moro”, Bari, Italy; 2 Department of Biomedical, Surgical and Dental Sciences, Milan University, Milan, Italy; 3 Department of Experimental Medicine, University of Salento, Lecce, Italy

**Keywords:** cephalometric analysis, craniofacial morphology, facial divergence, growing patients, masseter muscle, masticatory function, ultrasound assessment, vertical growth pattern

## Abstract

**Introduction:**

The relationship between vertical facial dimensions and morphological-functional features of the masseter muscle in growing patients is of increasing interest in orthodontics. Understanding these correlations may enhance diagnostic accuracy and guide treatment planning, particularly in subjects with altered vertical skeletal patterns.

**Materials and methods:**

Growing patients underwent two-dimensional cephalometric analysis and ultrasonographic evaluation of the masseter muscle. Cephalometric variables included SNA, SNB, ANB, SN-GoGn, FMA, AFH, and Go–Me, while ultrasonographic parameters comprised thickness, cross-sectional area (CSA), and volume of the masseter, both at rest and during contraction. Correlations were assessed using Pearson's coefficient, and multiple linear regression was applied to identify predictive associations. A *p*-value < 0.05 was considered statistically significant.

**Results:**

A significant correlation was found between vertical growth pattern and ultrasonographic characteristics of the masseter. Subjects with increased vertical facial dimensions exhibited reduced muscle thickness and volume (*p* < 0.05). Regression analysis confirmed associations between vertical cephalometric indices (SN-GoGn, FMA) and ultrasonographic parameters, with R^2^ values ranging from 0.30 to 0.45.

**Discussion:**

Findings suggest that vertical skeletal pattern influences masseter morphology, highlighting the role of masticatory musculature in craniofacial development and vertical discrepancies.

**Conclusions:**

Integrating ultrasonographic assessment of the masseter with cephalometric analysis may provide clinically relevant insights for orthodontic diagnosis and management in growing patients.

## Introduction

Craniofacial development is a dynamic and complex process influenced by numerous genetic, environmental, and functional factors. In orthodontics, understanding the relationship between skeletal morphology, muscular structure, and masticatory function is fundamental for accurate diagnosis and effective treatment planning, particularly in growing patients (1). Among the most widely investigated morphological parameters is the vertical facial dimension, a skeletal feature closely associated with craniofacial growth patterns (2, 3). Another key component involved in the regulation of lower facial development is the masseter muscle, whose functional activity and thickness have been shown to correlate with vertical facial growth type (4–9). Although several associations between masticatory muscle morphology and vertical facial dimension are well established, other aspects remain inconsistent or controversial in the current literature, with studies reporting variable findings regarding the strength and clinical significance of these relationships.

Vertical facial dimension, typically assessed through cephalometric parameters such as the mandibular plane angle (SN-GoGn), the facial angle (Frankfurt-Mandibular), or the anterior facial height (AFH), plays a critical role in skeletal pattern classification (9–13). It is commonly categorized into three main types: normovergent, hypovergent, and hypervergent. Hypovergent patients usually exhibit an anteriorly rotated mandible, a well-developed and tonically active masticatory musculature, a flatter occlusal plane, and a reduced vertical facial dimension (14–18). Conversely, hypervergent individuals tend to present with a posteriorly rotated mandible, a steeper occlusal plane, an increased vertical facial height, and a generally less developed masseter muscle (19–22). In the present study, vertical divergence was classified using Steiner and McNamara cephalometric criteria, applying the following cut-off values: SN–GoGn < 28° (hypodivergent), 28°–36° (normodivergent), >36° (hyperdivergent); FMA < 22°, 22°–28°, > 28°. These thresholds align with commonly accepted orthodontic diagnostic standards.

Numerous studies have highlighted the role of the masticatory muscles in craniofacial morphogenesis (23–26). The masseter muscle, in particular, plays a central role in chewing function and in the transmission of occlusal forces to the cranial skeleton (27–30). Several studies in the literature have suggested the existence of a bidirectional relationship between function and form: on the one hand, facial morphology may influence muscular development; on the other, the forces generated by the masticatory muscles may guide skeletal adaptation during growth. This concept is at the core of the functional matrix theory, which posits that bone structures adapt in response to the mechanical loads to which they are subjected. However, it is essential to underline that most available evidence describes correlational rather than causal relationships between muscle morphology and craniofacial growth, and causal interpretations should be made with caution (31).

Vertical growth pattern is influenced by multiple factors such as airway patency (mouth breathing, tonsillar hypertrophy), parafunctional habits, tongue posture, BMI and body build, sex, and skeletal maturity. These variables may influence muscular development and were not fully controlled in the present study (32–40). Recent systematic reviews and meta-analyses have further highlighted the heterogeneity of available findings, suggesting that discrepancies across studies may result from methodological variability, differences in imaging protocols, and age-related factors.

Assessing the structure and function of the masseter muscle in growing individuals presents a methodological challenge. Traditionally, this evaluation has been conducted using electromyography, ultrasonography, or advanced imaging techniques (MRI, CT). In particular, muscle ultrasonography has emerged as a non-invasive, reproducible, and radiation-free method, making it well-suited for studies in children and adolescents (41–44). Although bilateral measurements were collected, the study did not aim to analyse masseter asymmetry, and asymmetry was neither expected nor included in the statistical model. Concurrently, cephalometric analysis allows for precise evaluation of vertical facial characteristics through the identification of specific angular and linear measurements on lateral skull radiographs (45–52).

Clinically, the vertical facial pattern is a key parameter in orthodontic assessment and treatment planning (53). Hyperdivergent patients typically present increased vertical dimension, posterior mandibular rotation, and reduced masticatory efficiency, features often associated with lower masseter activity (54–61). Early recognition of these characteristics can support timely and targeted intervention during growth (62–78).

This study investigates the relationship between vertical facial dimension and masseter muscle morphology in a pediatric sample, exploring whether ultrasonographic muscle measurements correlate with cephalometric indicators of vertical skeletal pattern (79–83). By combining non-invasive ultrasonography with standardized cephalometric evaluation, the present work aims to clarify existing inconsistencies in the literature (83–87).

Although several studies have examined the interplay between muscular function and vertical facial morphology, current evidence remains partly contradictory, likely due to heterogeneous methodologies and confounding factors such as age, sex, or functional habits (88–95). The present study contributes to this debate by applying a homogeneous pediatric sample and a consistent acquisition protocol (96–100).

Overall, further understanding of how masseter characteristics relate to vertical skeletal pattern may help integrate muscular assessment into orthodontic diagnostics, supporting more individualized and functionally oriented treatment strategies (100–105).

The originality of the present research lies in the use of a homogeneous pediatric sample and the combined application of ultrasonographic and cephalometric methods following a standardized acquisition protocol.

## Materials and methods

The sample for this study consisted of 18 subjects (7 females and 11 males) who attended the Department of Dentistry at the Policlinico of Bari.

The study was conducted in accordance with the ethical principles outlined in the Declaration of Helsinki and was approved by Comitato Etico Locale IRCCS Istituto Oncologico “Gabriella Serio” (protocol n. 1010, date 03/12/2024; project code 1980 CEL—Muscle Function Analysis). The studies were conducted in accordance with the local legislation and institutional requirements. Written informed consent for participation in this study was provided by the participants' legal guardians/next of kin. It should be noted that the sample consisted exclusively of mesofacial and dolichofacial subjects. Hypodivergent subjects were not included in the present study, and this is now explicitly clarified to avoid misinterpretation. The absence of a hypodivergent group reduces the comparative value of the findings and represents a methodological limitation, as several key muscular adaptations described in the literature involve hypodivergent subjects. In this study, the terms normodivergent/mesofacial and hyperdivergent/dolichofacial are used interchangeably according to standard orthodontic definitions.

The mean age of the participants was 9.78 years (range: 6–15 years). Subjects were selected based on the following inclusion criteria:

Growing patients in the prepubertal developmental stage (CS1–CS3 cervical vertebral maturation stage);
Subjects in the mixed or permanent dentition phase;
Patients with normodivergent or hyperdivergent growth patterns based on cephalometric analysis.

Exclusion criteria included:

Absence of first molars, skeletal asymmetries, temporomandibular joint disorders, congenital craniofacial anomalies, history of facial trauma, and previous orthodontic treatment.

Each subject underwent a routine lateral cephalometric radiograph for diagnostic purposes. According to the mandibular plane angle [Frankfort horizontal plane/mandibular plane angle (FMA)], participants were divided into two subgroups based on their facial growth pattern:

Mesofacial group (11 subjects: 6 males, 5 females): 22°≤FMA ≤ 28°;
Dolichofacial group (7 subjects: 5 males, 2 females): FMA > 28°.

The thickness of the masseter muscle was measured in all subjects using ultrasonographic imaging. Scans were performed by a calibrated and experienced operator (F.I.) using a real-time scanner equipped with a linear array transducer. Intra-operator reliability was evaluated through repeated measurements on five randomly selected subjects, yielding excellent agreement (icc = 0.91; 95% ci: 0.85–0.96).

**Figure 1:**
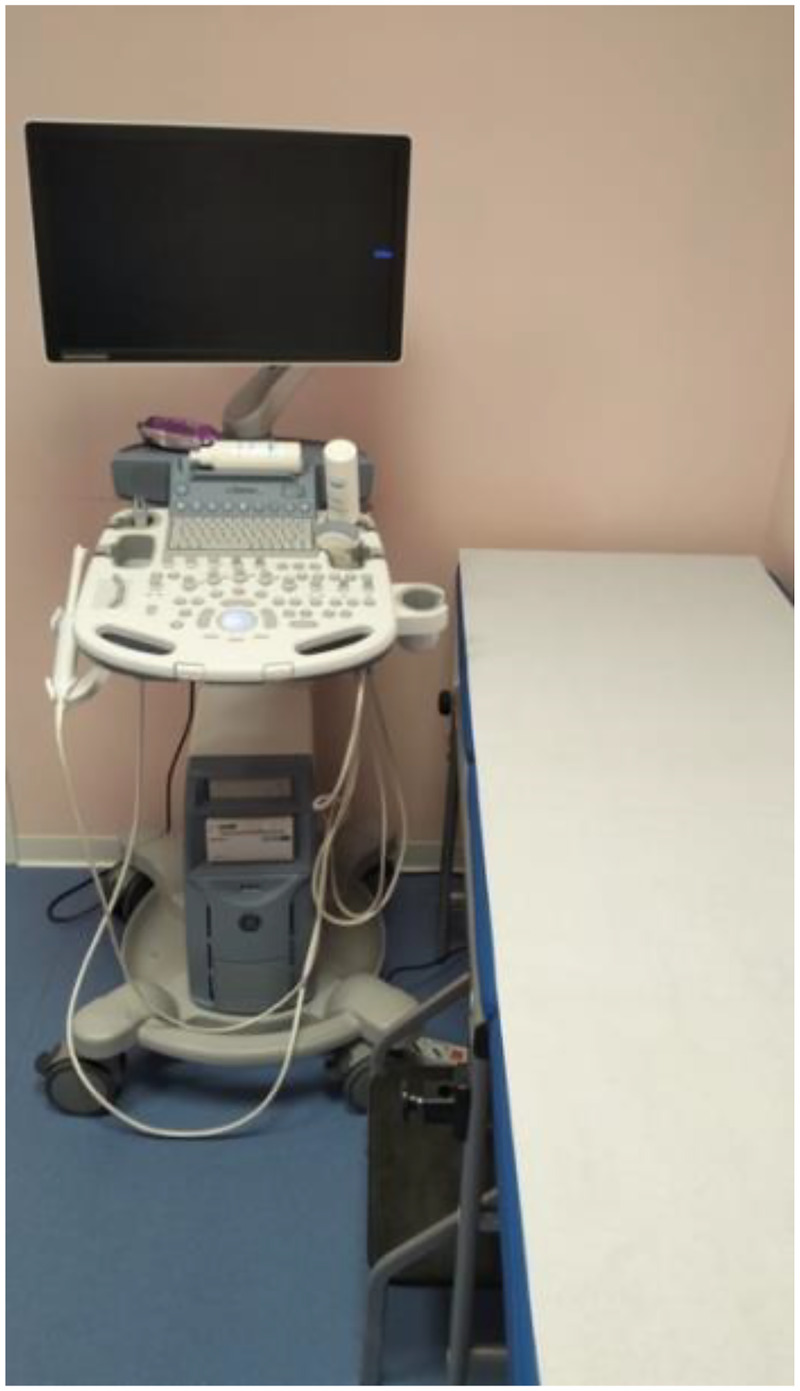
Device used for the study.

Real-time imaging of the masseter muscles was performed bilaterally using a high-frequency linear probe (9 MHz) connected to an ultrasound system (Siemens Acuson S2000) (Figure 1). The transducer was linked to an ultrasound scanner (Sony UPP 110), and muscle thickness was measured directly on the screen with a resolution of 0.01 mm. All images were acquired in B-mode and stored on film. Ultrasound parameters were standardized as follows: depth 20 mm, gain 52 db, dynamic range 72 db, frequency preset 9 mhz, to ensure reproducibility.

**Figure 2:**
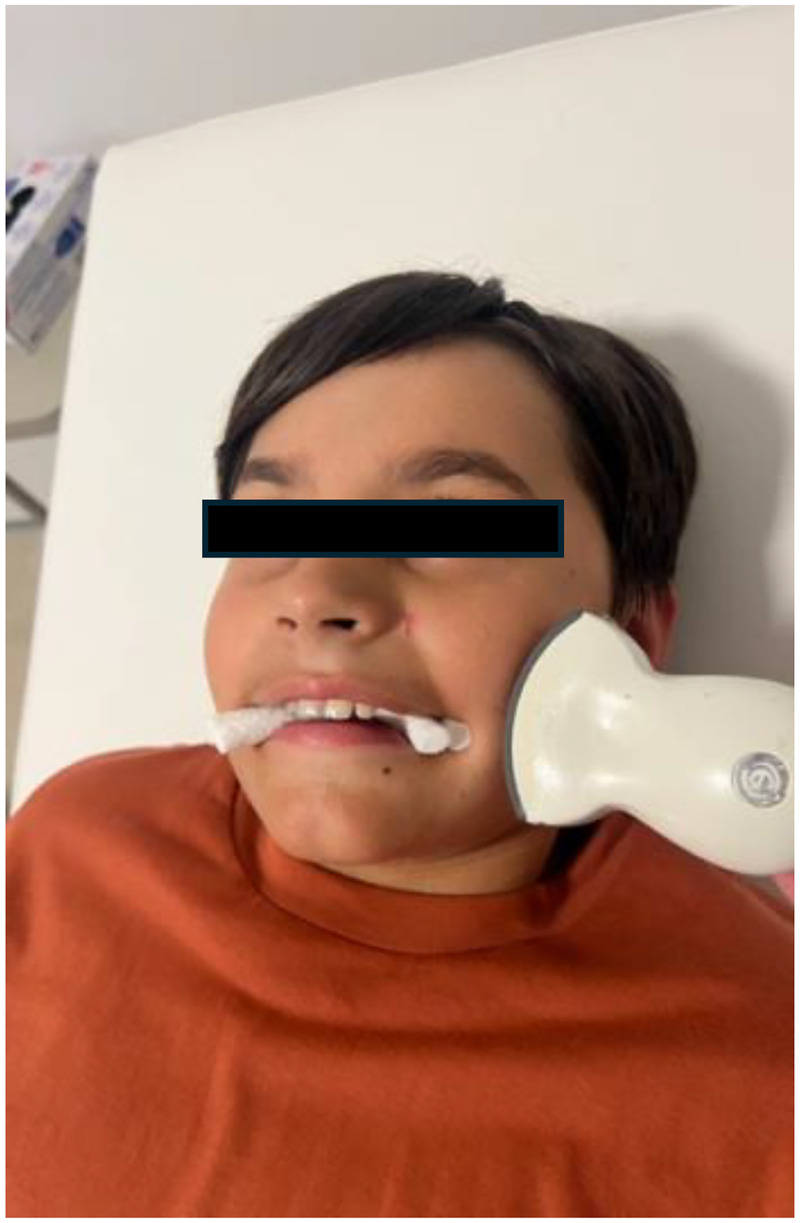
Ultrasound examination of the muscle (N.M., 9 y.o., male).

A water-based gel was applied to the probe to avoid tissue compression and irritation. During imaging, the transducer was held perpendicular to the skin surface, taking care not to apply excessive pressure, which could affect measurement accuracy (Figure 2).

To avoid overestimation due to oblique scanning of the masseter, the transducer angle was adjusted to achieve the optimal echogenic image of the mandibular ramus. Once the ramus appeared as a sharp, white linear contour on the screen, the transducer was considered to be perpendicular. To enhance contrast between the muscle and subcutaneous tissue, subjects were asked to clench and relax their muscles alternately.

The measurement site corresponded to the thickest portion of the masseter muscle, near the occlusal plane, approximately midway between the medial and lateral borders of the ramus and halfway between the zygomatic arch and the gonial angle. The measurement site was located approximately 15–20 mm above the gonial angle and parallel to the occlusal plane, with final positioning verified via real-time imaging. Two cotton rolls were placed bilaterally in the molar region, and patients were instructed to bite lightly to hold them in position.

**Figure 3:**
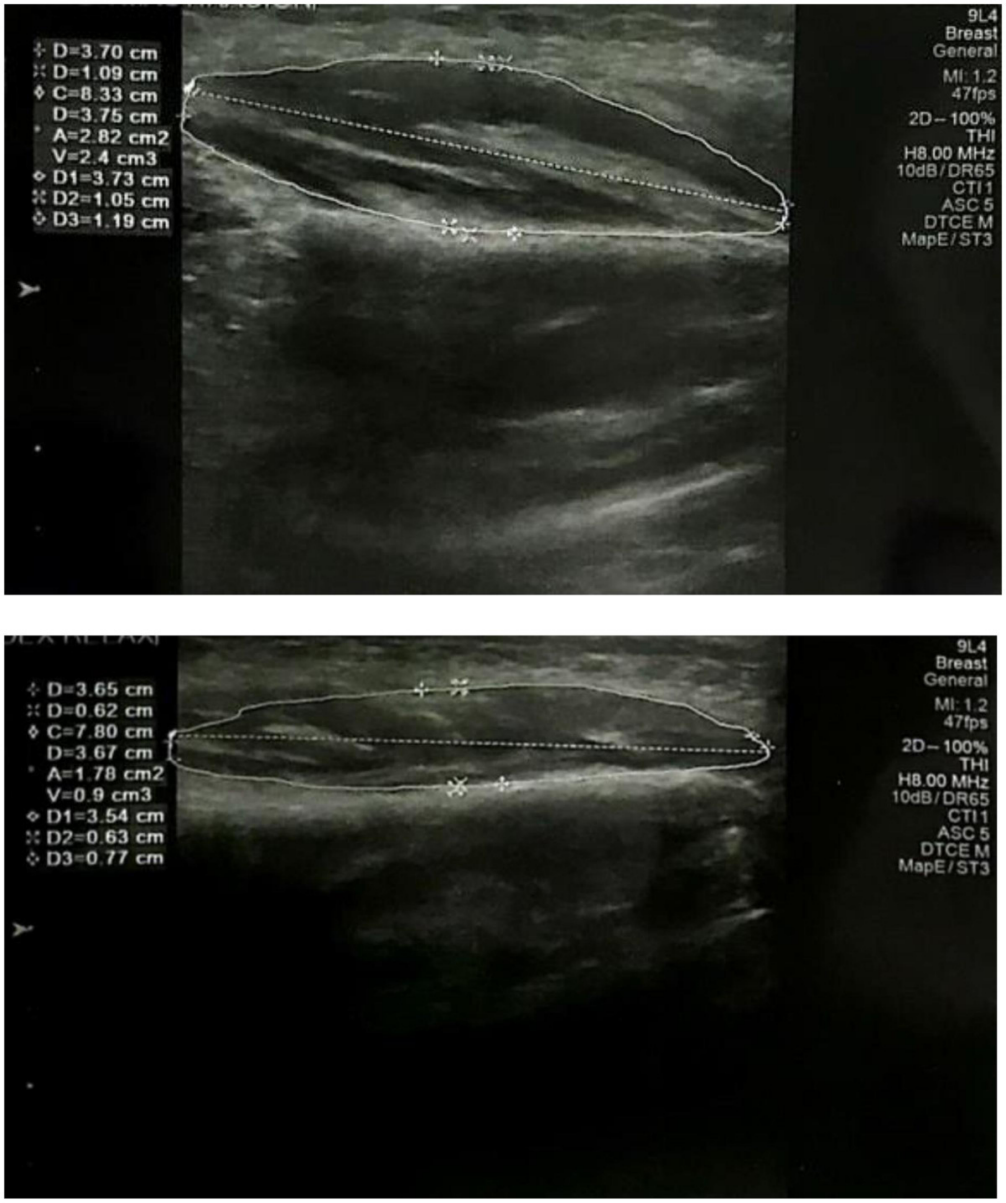
Ultrasound image of the masseter muscle.

Images and measurements were obtained bilaterally with the subject in a supine position and the head oriented straight. The supine position was selected to improve patient stability during scanning; however, differences from natural head posture should be considered, as seated acquisition is generally recommended. Measurements were taken in two functional conditions: (1) during light occlusion, with the muscle relaxed, and (2) during maximum clenching, with the masseter muscle contracted (Figure 3). Masseter thickness at rest was recorded without tooth contact; the light bite was used exclusively to stabilize the cotton rolls during contraction.

Muscle length, thickness, cross-sectional area, and volume were recorded. The latter two parameters were automatically calculated by the device's software (Figure 3). All ultrasonographic scans were performed using the same equipment and by the same expert in radiologic diagnostics, to minimize intra-operator variability.

**Figure 4:**
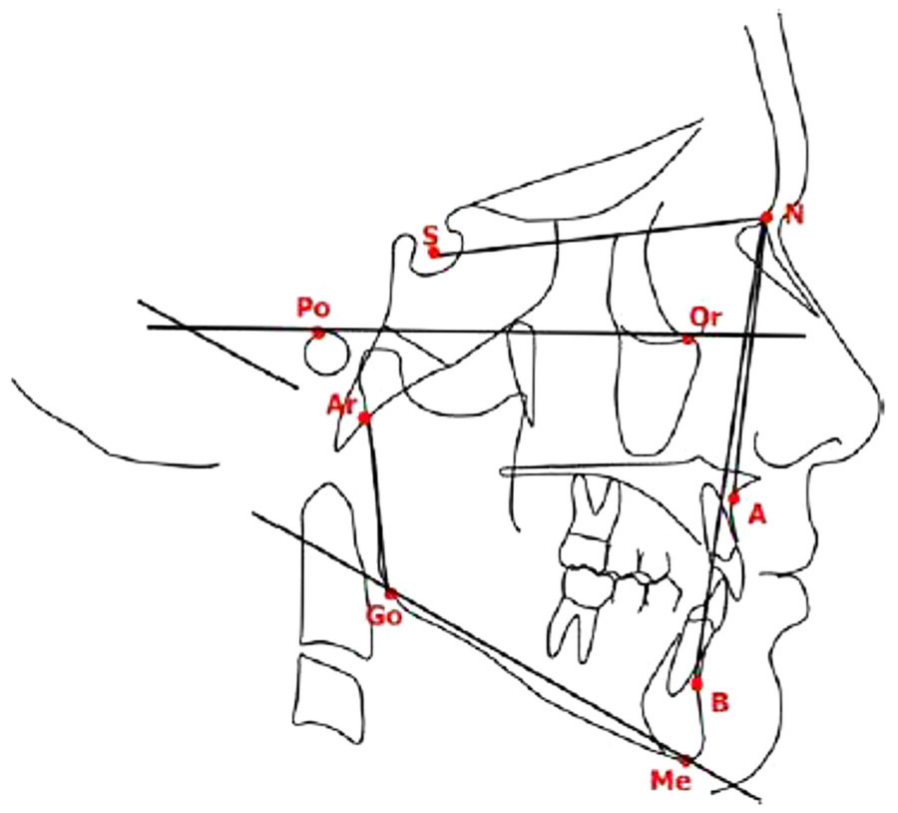
Cephalometric analysis performed for each patient.

Each subject also underwent a lateral cephalometric radiograph in centric occlusion at the time of the ultrasound scan. Cephalometric tracings were performed by a single operator to minimize bias, utilizing the Deltadent software (Outside Format, Meola Fabio, Via Circonvallazione D 28, 26025 Pandino, CR, Italy). Facial divergence was assessed by drawing the Frankfort plane and the mandibular plane (Go–Me), calculating the FMA angle according to the Tweed triangle. Additional standard angular measurements included: SNA (point A to Sella–Nasion), SNB (point B to Sella–Nasion), ANB (point A to Nasion to point B), and the gonial angle (posterior and lower borders of the mandible) (Figure 4).

## Statistical analysis

Data analysis was performed using IBM® SPSS® Statistics Version 20. A database was constructed using numerical coding in compliance with the software's requirements. Two types of analyses were performed:

Descriptive analysis, including the calculation of mean, median, mode, variance, standard deviation, and quartiles for continuous variables, as well as proportions with 95% confidence intervals for nominal variables.
Inferential analysis, primarily conducted through bivariate methods. Two-tailed tests were performed with a Type I error probability set at 5%.

The following statistical tests were applied:

Student's *t*-test for comparisons between continuous and nominal variables;
Linear regression analysis and *F*-test to examine associations between continuous variables;
Calculation of the coefficient of determination (R^2^) to assess the strength of correlation between continuous variables.

Both regression and correlation analyses were used to determine the contribution of multiple independent variables to the dependent variable, which represents the primary outcome of this study. The dependent variable used in regression analyses was masseter length, identified as the only parameter showing significant between-group differences.

The small sample size increases the risk of type II error and may limit the power of regression analyses. Effect sizes (Cohen's d, Pearson's r) and 95% confidence intervals were calculated to assess the magnitude and reliability of results. Standard interpretation thresholds were applied as recommended in the literature (Pearson's r: small = 0.20, medium = 0.40, large = 0.70; Cohen's d: small = 0.10, medium = 0.40, large = 0.90), in accordance with dental methodological guidelines (106). Findings with small effect sizes and non-significant *p*-values were considered not clinically meaningful.

## Results

Descriptive statistics for the cephalometric measurements, ultrasonographic measurements, and the results of statistical analyses classified according to the vertical growth pattern are listed in Table 1. No significant differences were found in the sagittal measurements between subjects with different vertical growth patterns (SNA and ANB). However, SNB and the gonial angle showed a significant increase from the mesofacial to the dolichofacial group.

**Table 1:** Significant between-group differences in masseter and cephalometric measurements.

Variable	Mesofacial mean ± SD	Dolichofacial mean ± SD	Difference (M2–M1)	Effect size (d)	95% CI
Masseter length (right, rest) (mm)	35.82 ± 1.60	39.00 ± 3.10	+3.18	1.39	0.70–5.66
Masseter length (right, rest; dx & sn) (mm)	35.57 ± 1.27	38.48 ± 3.20	+2.91	1.32	0.42–5.40
Masseter length (contraction; dx & sn) (mm)	35.27 ± 1.65	36.70 ± 1.08	+1.43	0.98	0.17–2.69
Masseter volume (right, rest) (mm^3^)	10.36 ± 2.06	14.00 ± 4.39	+3.64	1.16	0.17–7.11
SNB (°)	77.70 ± 2.73	81.07 ± 4.54	+3.37	0.96	−0.36–7.10
Gonial angle (°)	129.14 ± 1.64	134.73 ± 3.94	+5.59	2.04	2.51–8.67

No significant differences were found between mesofacial (Right Thickness: 7.28 mm, Left Thickness: 9.65 mm, Right Area: 18.53 mm^2^, Left Area: 24.18 mm^2^, Right Volume: 10.40 mm^3^, Left Volume: 18.13 mm^3^) and dolichofacial subjects (Right Thickness: 7.54 mm, Left Thickness: 9.92 mm, Right Area: 22.35 mm^2^, Left Area: 26.82 mm^2^, Right Volume: 13.35 mm^3^, Left Volume: 20.42 mm^3^) for masseter thickness during both relaxation and contraction.

Significant differences were found between mesofacial (Right Length: 35.57 mm, Left Length: 35.27 mm) and dolichofacial (Right Length: 38.48 mm, Left Length: 36.7 mm) subjects.

When subjects were classified according to different FMA values, ultrasonographic measurements showed a negative correlation between masseter muscle thickness and facial divergence (FMA°) during contraction (Table 2). However, due to the small sample size, the values were not statistically significant.

**Table 2:** Correlation between muscle measurements and FMA angle.

Ultrasonographic measurement	Pearson r	*p*-value	Interpretation
Thickness (R)	0.009	0.97	Negligible
Thickness (M)	−0.10	0.69	Small
Length (R)	0.29	0.22	Small–medium
Length (M)	0.37	0.13	Medium
Area (R)	0.22	0.37	Small
Area (M)	0.095	0.70	Negligible–small
Volume (R)	0.26	0.28	Small
Volume (M)	0.085	0.73	Negligible

## Discussion

Human facial growth is influenced by both genetic and environmental factors, with soft tissues and muscular function acting as important modulators of skeletal development (107, 108). Ultrasonographic muscle thickness and volume reflect morphological characteristics and correlate with functional capacity, but they do not directly quantify muscular activity, particularly in pediatric populations (109). Although the masseter muscle contributes to transverse and vertical mandibular development through mechanical loading, it remains unclear whether muscular forces primarily drive craniofacial growth or whether muscle morphology adapts secondarily to skeletal configuration during growth (110–113). Consequently, the associations observed in the present study, as well as in most of the available literature, should be interpreted as correlational rather than causal (114–116).

The present findings are generally consistent with previous studies reporting a negative relationship between vertical facial dimension and masseter size, with reduced muscle thickness and volume typically observed in individuals with increased facial height (117–119). This pattern has been interpreted as the result of adaptive remodeling or relative disuse of the masticatory muscles in hyperdivergent subjects, rather than as a primary etiological factor of vertical facial growth (120–122). However, direct comparison with the most robust literature is limited by the absence of hypodivergent (brachyfacial) subjects in our sample, a group that usually exhibits the greatest masseter development and represents an essential reference for interpreting muscular adaptations across facial biotypes (123–125).

Across both mesofacial and dolichofacial groups, masseter thickness, surface area, and volume increased during contraction, while muscle length decreased, reflecting normal physiological behavior (126). Among all ultrasonographic parameters, muscle length was the only variable showing statistically significant differences between groups, with dolichofacial subjects exhibiting greater masseter length both at rest and during contraction. This finding partially contrasts with studies that emphasize muscle thickness and volume as the primary discriminating parameters (127, 128), and suggests that longitudinal muscle adaptation may be particularly sensitive to vertical skeletal divergence in growing individuals.

An unexpected finding was the significantly greater resting volume of the right masseter muscle in dolichofacial subjects. This result contrasts with most previous reports describing reduced masseter dimensions in long-faced individuals (129). Several explanations may be proposed. First, this isolated unilateral difference may reflect functional asymmetry, which is relatively common in children and does not necessarily correspond to clinically relevant imbalance. Second, it may represent adaptive morphological remodeling rather than increased functional efficiency, particularly in the absence of consistent bilateral differences or corresponding increases during contraction. Finally, the operator-dependent nature of ultrasonography and the small sample size may have contributed to measurement variability. For these reasons, this finding should be interpreted with caution.

From a biomechanical and neurophysiological perspective, the observed differences may reflect adaptive mechanisms of the masticatory muscles in response to vertical skeletal configuration. Increased muscle length in dolichofacial subjects may represent an adaptation to altered mandibular geometry and reduced mechanical advantage, involving changes in muscle–tendon unit behavior and neuromuscular recruitment patterns during growth (130). Conversely, greater muscle thickness and volume described in brachyfacial individuals in previous studies may reflect higher mechanical loading and more efficient force transmission to the craniofacial skeleton (131). These observations support the concept of a bidirectional interaction between muscle and bone, without implying a unidirectional causal relationship.

Correlations between ultrasonographic measurements and FMA° were generally low and statistically non-significant. This finding is not unexpected given the small sample size and the multifactorial nature of vertical facial growth. Importantly, interpretation based solely on *p*-values may be misleading. In the present study, several parameters—particularly masseter length and gonial angle—showed medium to large effect sizes, suggesting potential clinical relevance despite limited statistical power.

## Clinical implications

From a clinical standpoint, these findings suggest that assessment of the masseter muscle—particularly its longitudinal behavior—may provide complementary information to traditional cephalometric analysis in growing patients. Ultrasonographic evaluation of masticatory muscles could therefore contribute to a more comprehensive functional diagnosis and support individualized orthodontic treatment planning in subjects with vertical growth tendencies.

## Limitations

This study presents several limitations. The small sample size reduces statistical power and increases the risk of type II error. The absence of hypodivergent subjects limits full-spectrum comparison across facial types. Potential confounding variables such as sex, biological maturation, BMI, airway conditions, and functional habits were not controlled. Ultrasonographic measurements, although reliable, remain operator-dependent, and the use of the supine position differs from natural head posture recommended in some protocols.

## Conclusions

Masseter muscle morphology shows correlational associations with vertical facial pattern in growing patients.
Muscle length appears more sensitive to vertical skeletal divergence than thickness or volume.
Effect-size analysis indicates that some differences may be clinically meaningful despite non-significant *p*-values.
Muscular assessment may enhance orthodontic diagnostics when interpreted within acknowledged methodological limitations.

## Data Availability

The raw data supporting the conclusions of this article will be made available by the authors, without undue reservation.
